# Design, Modeling, and Testing of a Novel XY Piezo-Actuated Compliant Micro-Positioning Stage

**DOI:** 10.3390/mi10090581

**Published:** 2019-08-31

**Authors:** Quan Zhang, Jianguo Zhao, Xin Shen, Qing Xiao, Jun Huang, Yuan Wang

**Affiliations:** 1 School of Mechatronic Engineering and Automation, Shanghai University, Shanghai 200072, China; 2 National Research Center of Pumps, Jiangsu University, Zhenjiang 212013, China; 3 College of Communication Engineering, Army Engineering University of PLA, Nanjing 210007, China

**Keywords:** compliant mechanism, micro-positioning stage, Castigliano’s second theorem, beam constrained model, bridge-type amplification mechanism

## Abstract

A novel decoupled XY compliant micro-positioning stage, based on a bridge-type amplification mechanism and parallelogram mechanisms, is designed in this paper. Analytical models of the bridge-type amplification mechanism and parallelogram mechanisms are developed by Castigliano’s second theorem and a Beam constrained model. The amplification ratio, input stiffness, and output stiffness of the stage are further derived, based on the proposed model. In order to verify the theoretical analysis, the finite element method (FEM) is used for simulation and modal analysis, and the simulation results indicate that the errors of the amplification ratio, input stiffness, and output stiffness of the stage between the proposed model and the FEM results are 2.34%, 3.87%, and 2.66%, respectively. Modal analysis results show that the fundamental natural frequency is 44 Hz, and the maximum error between the theoretical model and the FEM is less than 4%, which further validates the proposed modeling method. Finally, the prototype is fabricated to test the amplification ratio, cross-coupling error, and workspace. The experimental results demonstrate that the stage has a relatively large workspace, of 346.1 μm × 357.2 μm, with corresponding amplification ratios of 5.39 in the X-axis and 5.51 in the Y-axis, while the cross-coupling error is less than 1.5%.

## 1. Introduction

With the rapid development of microelectromechanical systems (MEMS), nanotechnology, and precision engineering, the demands on precision positioning technology have been increasing. Conventional rigid positioning stages composed of transmission gears and rigid joints are no longer suitable, due to the backlash and friction. New micro/nano-positioning stages based on compliant mechanisms has attracted the attention of many scholars, due to their characteristics of no friction, no backlash, no lubrication, and ease of fabrication [1]. Moreover, it is vital to employ actuators with high resolution for precision positioning. Commonly used actuators in precision driving fields can be classified as follows: piezoelectric actuators (PEAs), voice coil motors (VCMs), electromagnetic actuators, magnetostrictive actuators, shape memory alloy (SMA) actuators, and electrostatic actuators, among others. [2,3,4,5]. Due to the requirements of the external magnetic field, which usually makes the structure of the system hard to miniaturize, electromagnetic actuators and magnetostrictive actuators have rarely been adopted in micro-positioning stages. Shape memory alloy actuators have complex force-electric-thermal coupling relationships, making it difficult to achieve precise control when using them. Common electrostatic actuators usually require a large driving voltage to achieve a desired output displacement. However, a large driving voltage will tend to cause electrostatic breakdown, affecting reliability [6]. Thus, PEAs and VCMs have typically been adopted to actuate compliant micro/nano positioning stages [7,8,9,10,11]. The advantages of PEAs are high motion resolution, large output force, and fast response; however, the output displacement is usually small, with only a 0.1–0.2% range of its own length, which does not meet the requirements of some applications requiring compact size and large stroke [12]. Moreover, the inherent non-linear effects of these actuators, such as hysteresis and creep, also decrease the motion accuracy and positioning resolution of the micro-positioning system [13]. VCMs have the advantages of simple structure, small volume, high frequency response, and high precision, but their output force is small, making them difficult to apply in stages with large input stiffness and compact size [14]. Compared with VCMs, piezoelectric transducers have also been widely applied in active vibration control and precision driving fields [15,16]. Thus, the PEA was selected as the driving component in this study.

To overcome the difficulties caused by the small stroke of the PEAs, a compliant displacement amplification mechanism is usually introduced, between the actuator and the micro-positioning stage, to improve the output displacements of the system. Commonly used displacement amplification mechanisms include lever [17,18], bridge-type [19,20], and Scott–Russell [21,22] amplification mechanisms. Awtar et al. [23] presented a two degrees of freedom (2-DOFs) planar X–Y flexure mechanism based on flexible beams. The stiffness characteristics and cross-coupling errors of the stage were investigated. Due to the low cross-coupling characteristics of the double parallelogram mechanism, kinematic decoupling was almost achieved. A novel 2-DOFs micro-positioning stage has been developed by Wang [24] using corrugated flexible (CF) beams, instead of notched flexible hinges and rigid beams. The stiffness characteristics of the stage were analyzed by the stiffness matrix method and the dimensions of the stage were optimized with the aim of a higher off-axis/axial stiffness ratio and larger motion stroke. A 1-DOF stage with a sandwich-like structure has been devised by Wu [25] for micro/nano-positioning in the vertical direction. The actuator was placed in the middle plane and two orthogonal bridge-type amplification mechanisms were placed in the other two planes, which made it a sandwich structure. Such a design made the structure of the stage more compact. Tang et al. [26] designed a parallel X–Y micromanipulator using compound parallelogram mechanisms based on flexible hinges and lever amplification mechanisms. Theoretical modeling and finite element simulation of the static and dynamic characteristics of the stage were investigated, and the results indicated that the theoretical model and the finite element model had high consistency. Li et al. [27] combined compound bridge-type amplification mechanisms and compound parallelogram mechanisms based on flexible beams to design a completely decoupled micro-motion stage. The stiffness characteristics and amplification ratio were analyzed by the compliance matrix method. An experiment was set up, and the results showed that the cross-coupling error of the stage was less than 2%. Wu et al. [28] proposed a 2-DOFs nano-positioning stage with stacked structure. A bridge-type amplification mechanism was employed to amplify the output displacement of the PEA and a new decoupling mechanism based on compound parallelogram flexures was developed. FEA-based optimization aimed at maximizing the natural frequency was also conducted. The experimental results indicated that the fundamental frequencies of the X-axis and Y-axis were 64.03 Hz and 54.75 Hz, and that the workspace of the stage was 212.48 × 219.24 μm^2^ with a resolution of 7 nm. Lin et al. [29] analyzed the characteristics of a 6-DOFs compliant stage using a bond graph approach, by combining a pseudo-rigid-body (PRB) model and elastic beam theory. The kinematic performance, dynamic responses, and load capacity were investigated. Qin et al [30] designed a 3-DOFs planar compliant manipulator based on the improved Scott–Russell mechanism, and the inverse kinematics model, dynamics model, and the workspace of the manipulator was developed.

In addition, many hybrid amplification mechanisms, consisting of commonly used amplification mechanisms, have recently been developed [12,31,32,33,34]. Zhu et al. [31] proposed a novel displacement amplification mechanism, which was composed of two Scott–Russell mechanisms and a half bridge-type amplification mechanism. Combined with the positioning stage, a decoupled 2-DOFs nano-positioning stage was designed, and the displacement amplification ratio, stiffness model, maximum stress, and dynamic characteristics of the stage were analyzed. Zhang et al. [33] proposed a 3-DOFs spatial compliant micro-positioning stage which was able to move along the X, Y, and Z axes. The bridge-type amplification mechanism and the lever amplification mechanism were connected in series to form a two-stage amplification mechanism. Experimental results demonstrated that the cross-coupling error along X, Y, and Z axes was less than 5%. The above achievements were mostly combined by displacement amplification mechanisms and compound parallelogram mechanisms to devise a positioning stage, and a combination of displacement amplification mechanism and double parallelogram mechanisms was rarely used. Hence, in this paper, a decoupled 2-DOFs planar compliant micro-positioning stage was designed using bridge-type amplification mechanisms, as well as double-parallelogram and compound-parallelogram mechanisms, to achieve high decoupling characteristics with a relatively long stroke.

The rest of this paper is organized as follows: The mechanical design and working principle of the compliant positioning stage are introduced in Section 2; In Section 3, mathematical models, including a stiffness model of the parallelogram mechanism, a model of bridge-type amplification mechanism, and a stiffness model of the positioning stage, are established by Beam constrained model and Castigliano’s second theorem. In Section 4, the finite element simulation is presented. In Section 5, experimental verification is conducted. Finally, conclusions are provided in Section 6.

## 2. Mechanical Design and Working Principle of Compliant Positioning Stage

**Figure 1:**
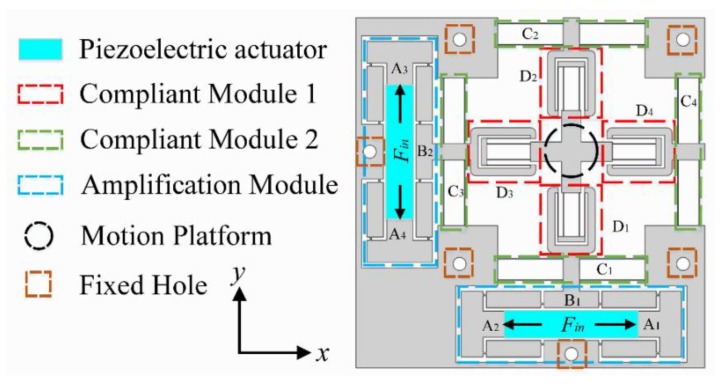
Mechanical design of compliant positioning stage.

The compliant positioning stage, with a symmetrical design, is mainly composed of two bridge-type amplification mechanisms (Amplification Module), four double parallelogram mechanisms (Compliant Module 1), four compound parallelogram mechanisms (Compliant Module 2), and a motion platform, as shown in Figure 1. Based on the aforementioned discussion of the different actuators used in precision driving fields, PEAs were selected as the driving components and installed into the bridge-type amplification mechanisms.

**Figure 2:**
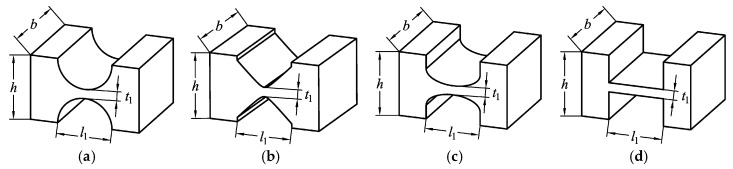
Four commonly used flexure hinges: (**a**) Circular notch, (**b**) hyperbolic notch, (**c**) elliptical notch, and (**d**) right angle notch.

**Figure 3:**
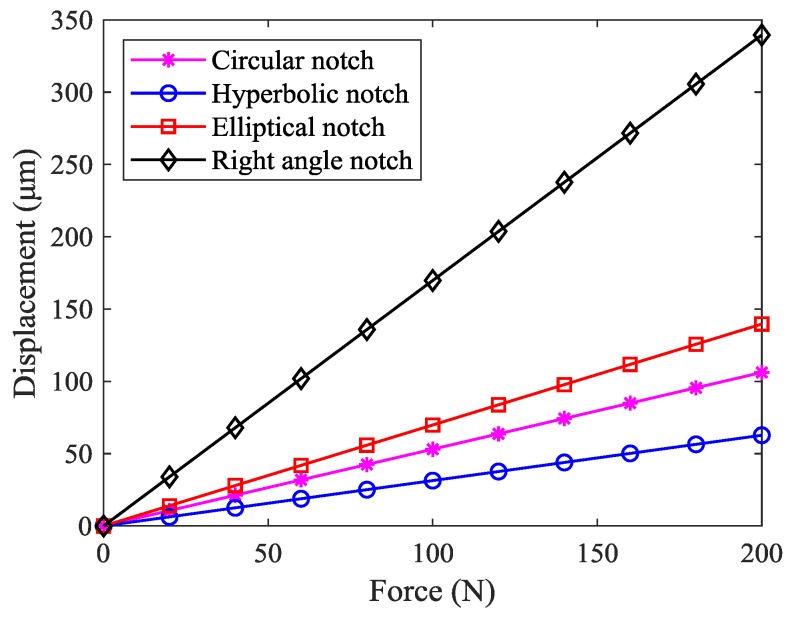
Force-displacement relationship of four common flexure hinges.

In the literature, various types of flexible hinges with different notches have been developed [35,36,37,38,39], including the commonly used circular notch type, hyperbolic notch type, elliptical notch type, and right angle notch type, as shown in Figure 2. In order to obtain the stiffness characteristics, the force-displacement relationship of four common flexure hinges was analyzed by ANSYS. It should be noted that the dimension parameters and material of each flexure hinge are the same in the finite element analysis. The results, as shown in Figure 3, indicate that the displacement of the right angle notch flexible hinge was the largest when an equivalent force was applied. The parallelogram mechanism constructed by flexible beams provided a larger linear deformation region than that constructed by flexible hinges and rigid beams [40]. In addition, it is easier to achieve higher accuracy when machining straight contours, rather than curved contours. For large motion stroke and ease of fabrication, the bridge-type amplification mechanism and parallelogram mechanism were constructed using right angle notch flexible hinges with lumped compliance and flexible beams with distributed compliance, respectively. 

**Figure 4:**
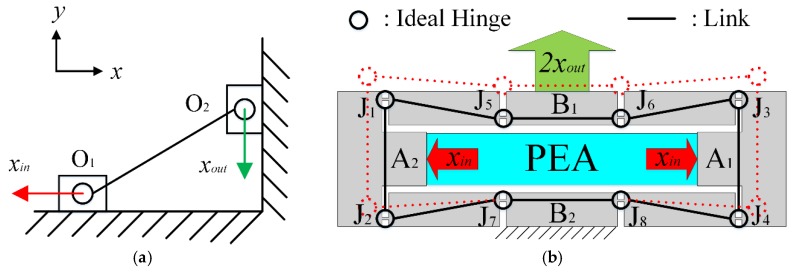
PRB model of the bridge-type amplification mechanism: (**a**) Working principle, and (**b**) overall scheme. (PRB model: pseudo-rigid-body model, PEA: piezoelectric actuator)

**Figure 5:**
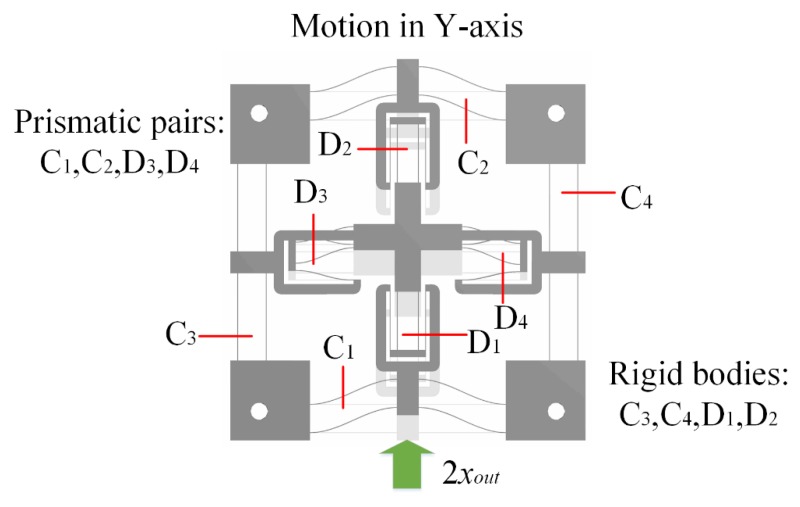
Working principle of parallelogram mechanisms.

Due to the symmetry of the stage, only motion in the Y-axis of the stage is taken as an example to demonstrate the working principle. The working principle and PRB model of the bridge-type amplification mechanism are illustrated in Figure 4. As the bridge-type amplification mechanism is composed of four symmetrical branches, one of the branches is taken to present the working principle, and the equivalent model is shown in Figure 4a. When an input displacement *x_in_* is applied to the point O_1_ along the X-axis, a displacement *x_out_* along the Y-axis is generated at the point O_2_. As can be observed from Figure 4b, the motion state of the branch J_2_J_7_ is similar to that of O_1_O_2_. The only difference is that the point J_7_ is a fixed hinge, so point J_2_ moves the displacement of *x_out_* along the positive direction of Y-axis while moving along X-axis. Similarly, when point J_1_ moves along the negative direction of X-axis, the output displacement *x_out_* along the positive direction of Y-axis is generated at point J_5_. However, as point J_2_ raises the initial position of point J_5_ by *x_out_*, the actual output displacement of point J_5_ is 2*x_out_*. The output displacement of point J_6_ is the same as point J_5_, due to the symmetrical structure. It should be noted that the values of the displacement along X-axis are equal and the directions are opposite; therefore, link B_1_ only moves along the Y-axis. Thus, when the inputs A_1_ and A_2_ of the bridge-type amplification mechanism are driven by a force *F_in_* along the X-axis, the input displacements *x_in_* will be applied in the positive and negative directions of the X-axis, respectively. Simultaneously, an output displacement of 2*x_out_* in the positive direction of the Y-axis will be produced at link B_1_. Due to the high axial stiffness and low transverse stiffness of flexible beams, the compound parallelogram mechanisms C1 and C2 and double parallelogram mechanisms D3 and D4 can be assumed to be prismatic pairs of the motion. While the compound parallelogram mechanisms C3 and C4 and double parallelogram mechanisms D1 and D2 can be assumed to be rigid bodies, the linear motion of the motion platform is finally achieved through motion transmission, as shown in Figure 5. The working principle is similar in the X-axis, but the prismatic pairs and rigid bodies are exchanged. Thus, 2-DOFs of motion (i.e., in the X and Y directions) are obtained. Double parallelogram mechanisms and compound parallelogram mechanisms with small cross-coupling error are adopted in the design, which can effectively attenuate the cross-coupling displacement.

## 3. Mathematical Model of the Positioning Stage

### 3.1. Stiffness Model of the Parallelogram Mechanism

**Figure 6:**
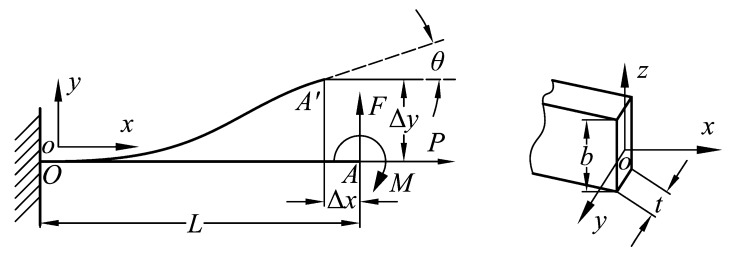
Large deflection of the flexible beam.

The double parallelogram mechanism and the compound parallelogram mechanism are both composed of flexible beams. In general, the flexible beam is in a state of large deflection and, so, the analysis of the flexible beam involves complicated non-linear factors. The models for the non-linear deflection of a beam are: The PRB model (including the improved PRB model, which can be utilized to analyze flexible beams with inflection points) [41,42,43], the Beam constraint model, the non-linear finite element method, and the elliptic integral method. When the transverse displacements of the flexible beam are as small as the thickness of the beam, the non-linearity caused by the force equilibrium conditions becomes significant, due to the load-stiffening and elastokinematic effects. While PRB models capture load-stiffening, their inherent lumped-compliance assumption precludes elastokinematic effects (for more details, see [44]). High calculation accuracy can be obtained by the non-linear finite element and ellipse integral methods, while a large number of nodes and complex integral calculation make such methods unfavorable for early design. In contrast, high precision with a simple calculation can be achieved using the beam constraint model when the beam deformation does not exceed 10% of the beam length, and this model is a general method which can calculate the deformation of flexible beams regardless of whether there are inflection points [45]. Therefore, the Beam constrained model was applied to model the flexible beams in this paper. As the parallelogram mechanisms are composed of flexible beams, a single flexible beam was first modeled. The structural parameters and deformation are shown in Figure 6, where *F*, *P*, and *M* are the lateral and axial forces and moment of the flexible beam, respectively, and *b*, *t*, and *L* are the width, thickness, and length of the flexible beam, respectively.

The beam constraint model can be expressed as:

(1) $$\left[\begin{array}{c} f\\ m \end{array}\right]=\left[\begin{array}{cc} a & c\\ c & b \end{array}\right]\left[\begin{array}{c} \delta_{y}\\ \theta \end{array}\right]+p\left[\begin{array}{cc} e & h\\ h & g \end{array}\right]\left[\begin{array}{c} \delta_{y}\\ \theta \end{array}\right]$$

(2) $$\delta_{x}=\frac{p}{d}+\left[\begin{array}{cc} \delta_{y} & \theta \end{array}\right]\left[\begin{array}{cc} i & k\\ k & j \end{array}\right]\left[\begin{array}{c} \delta_{y}\\ \theta \end{array}\right]+p\left[\begin{array}{cc} \delta_{y} & \theta \end{array}\right]\left[\begin{array}{cc} r & q\\ q & s \end{array}\right]\left[\begin{array}{c} \delta_{y}\\ \theta \end{array}\right].$$

The corresponding parameters in Equations (1) and (2) are shown in Table 1. We have:

(3) $$f=\frac{FL^{2}}{EI},m=\frac{ML}{EI},p=\frac{PL^{2}}{EI},$$

(4) $$\delta_{x}=\frac{\Delta x}{L},\delta_{y}=\frac{\Delta y}{L},T=\frac{t}{L},d=\frac{12}{T^{2}}.$$

**Figure 7:**
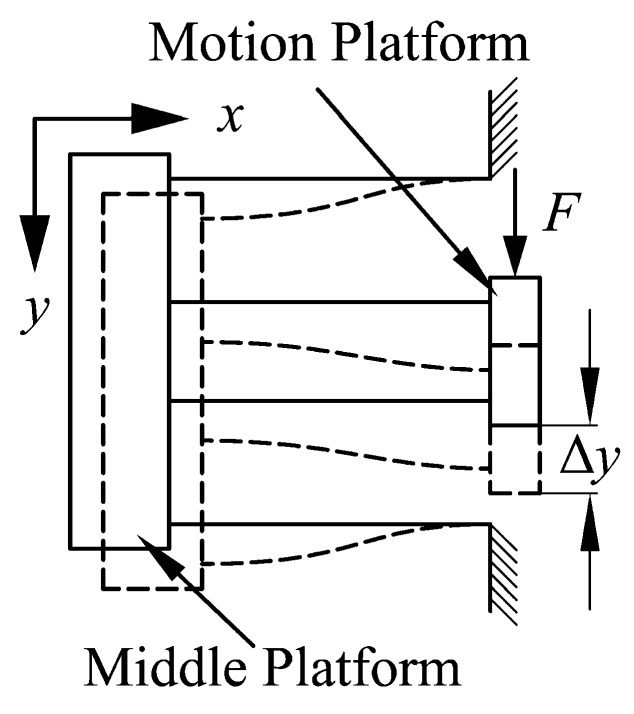
Deformation of the double parallelogram mechanism.

In the actual motion of the positioning stage, the deformation of the double parallelogram mechanism is shown in Figure 7. The same deformation of each flexible beam can be assumed, due to the same structure. Due to the two-stage deformation, when the moving platform moves Δ*y*, the displacement at the end of each beam is equal to Δ*y*/2. The middle platform can move along the X-axis during the motion; therefore, *P* can be approximated as equal to 0. Substituting *P* = 0 into Equation (1), the relationship between force and displacement for a double parallelogram mechanism can be derived as

(5) $$f=12\delta_{y}.$$

**Figure 8:**
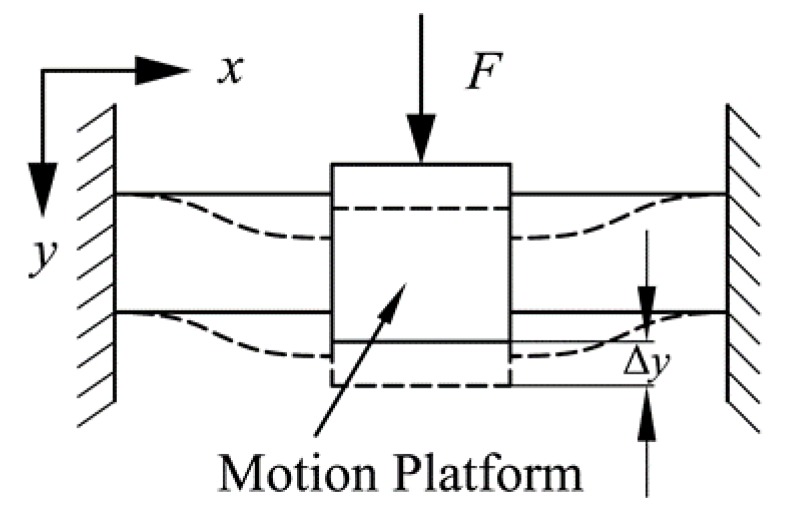
Deformation of the compound parallelogram mechanism.

The deformation of the compound parallelogram mechanism is shown in Figure 8. Each flexible beam can be considered to be a fixed guiding beam (i.e., Δ*x =* 0 and the tip deflection angle is *θ* = 0). Substituting Δ*x =* 0 and *θ* = 0 into Equations (1) and (2), the relationship between force and displacement of a compound parallelogram mechanism can be derived as:

(6) $$p=\frac{0.6\delta_{y}^{2}}{1/d+\delta_{y}^{2}/700}$$

(7) $$f=12\delta_{y}+1.2p\delta_{y}.$$

When the compound parallelogram mechanism moves along the *y*-axis, it can be considered as a parallel arrangement of four fixed guiding beams. Therefore, the driving force required to produce the same displacement becomes four times larger than that of a single flexible beam. The relationship between force and displacement of a compound parallelogram mechanism is formulated as:

(8) $$p=\frac{0.6\delta_{y}^{2}}{1/d+\delta_{y}^{2}/700}$$

(9) $$f=48\delta_{y}+4.8p\delta_{y}.$$

Substituting Equation (3) into Equations (5), (8), and (9), the lateral stiffness of the double parallelogram mechanism *K_DPM_* can be derived as

(10) $$K_{DPM}=\frac{12EI}{L^{3}}.$$

The lateral stiffness of the compound parallelogram mechanism *K_CBPM_* is calculated as:

(11) $$K_{CBPM}=\left(48+\frac{2.88\delta_{y}^{2}}{1/d+\delta_{y}^{2}/700}\right)\cdot \frac{EI}{L^{3}}.$$

### 3.2. Model of Bridge-type Amplification Mechanism

**Figure 9:**
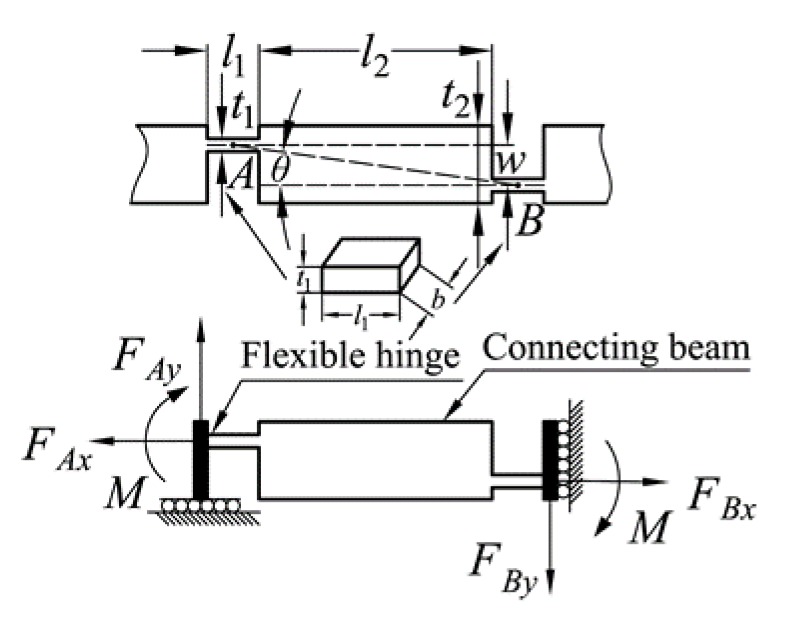
Mechanical analysis of the bridge-type amplification mechanism.

The bridge-type amplification mechanism consists of four symmetrical branches and, hence, only one of the branches is required to be mathematically modeled. The input and output ends are considered as rigid parts, due to the high stiffness. The force analysis and structural parameters of the remaining parts are shown in Figure 9. As the deformations of the two flexible hinges are basically the same, it can be assumed that the moments *M* of the two flexible hinges are equal. According to the force and moment balance equations, the following equations can be obtained:

(12) $$F_{Ax}=F_{Bx}=\frac{1}{2}F_{in}=f_{x},$$

(13) $$F_{Ay}=F_{By}=\frac{1}{2}F_{out}=f_{y},$$

(14) $$2M=f_{x}\cdot w-f_{y}\cdot \left(l_{1}+l_{2}\right).$$

The input and output displacements of the bridge-type amplification mechanism can be obtained by Castigliano’s second theorem. The branch of the bridge-type amplification mechanism is considered as a three-segment beam; that is, right angle flexible hinges are treated as short flexible beams.

(15) $$\begin{array}{ll} x_{in} & =2\int_{0}^{l_{1}}\frac{F_{l}(x)}{EA_{1}}\cdot \frac{\partial F_{l}(x)}{\partial f_{x}}+2\int_{0}^{l_{1}}\frac{M(x)}{EI_{1}}\cdot \frac{\partial M(x)}{\partial f_{x}}\\ & +\int_{l_{1}}^{l_{1}+l_{2}}\frac{F_{l}(x)}{EA_{2}}\cdot \frac{\partial F_{l}(x)}{\partial f_{x}}+\int_{l_{1}}^{l_{1}+l_{2}}\frac{M(x)}{EI_{2}}\cdot \frac{\partial M(x)}{\partial f_{x}} \end{array},$$

(16) $$\begin{array}{ll} x_{out} & =2\int_{0}^{l_{1}}\frac{F_{l}(x)}{EA_{1}}\cdot \frac{\partial F_{l}(x)}{\partial f_{y}}+2\int_{0}^{l_{1}}\frac{M(x)}{EI_{1}}\cdot \frac{\partial M(x)}{\partial f_{y}}\\ & +\int_{l_{1}}^{l_{1}+l_{2}}\frac{F_{l}(x)}{EA_{2}}\cdot \frac{\partial F_{l}(x)}{\partial f_{y}}+\int_{l_{1}}^{l_{1}+l_{2}}\frac{M(x)}{EI_{2}}\cdot \frac{\partial M(x)}{\partial f_{y}} \end{array},$$

where *A*_1_ and *A*_2_ are the axial cross-sectional areas of the flexible hinges and connecting beams, respectively; *I*_1_ and *I*_2_ are moment of inertia of the axial cross sections of the flexible hinges and connecting beams, respectively; and *E* is Young’s modulus. Uniform flexible beams have been investigated comprehensively over recent years [46,47]. However, the three-segment beam is a non-uniform flexure, which will be analyzed in the future. Here, to simplify the calculation, the same hypothesis as in the literature [48] is adopted, as follows:

(17) $$F_{l}(x)=f_{x},$$

(18) $$M(x)=\frac{1}{2}[f_{x}\cdot w-f_{y}\cdot (l_{1}+l_{2})].$$

Substituting Equations (15) and (16) into Equations (13) and (14), the results are further rearranged to obtain the following matrix expression:

(19) $$\left(\begin{array}{c} x_{in}\\ x_{out} \end{array}\right)=\left[\begin{array}{cc} C_{11} & C_{12}\\ C_{21} & C_{22} \end{array}\right]\cdot \left(\begin{array}{c} f_{x}\\ f_{y} \end{array}\right),$$

where *C*_11_, *C*_12_, *C*_21_, and *C*_22_ are the compliance matrix parameters of the bridge-type amplification mechanism, detailed as:

$$\begin{array}{c} C_{11}=\frac{2}{K_{l1}}+\frac{1}{K_{l2}}+\frac{w^{2}}{2K_{\theta 1}}+\frac{w^{2}}{4K_{\theta 2}},\;C_{12}=\frac{w(l_{1}+l_{2})}{2K_{\theta 1}}+\frac{w(l_{1}+l_{2})}{4K_{\theta 2}},\\ C_{21}=\frac{w(l_{1}+l_{2})}{2K_{\theta 1}}+\frac{w(l_{1}+l_{2})}{4K_{\theta 2}},\;C_{22}=\frac{{\left(l_{1}+l_{2}\right)}^{2}}{2K_{\theta 1}}+\frac{{\left(l_{1}+l_{2}\right)}^{2}}{4K_{\theta 2}}, \end{array}$$

where $K_{l1}$, $K_{l2}$, $K_{\theta 1}$, and $K_{\theta 2}$ are the axial tensile stiffnesses and rotational stiffnesses of the flexible hinges and connecting beams, respectively. As only the plane stiffness of the bridge-type amplification mechanism needs to be considered (according to reference [49]), the following simplified results are obtained:

$$K_{l1}=\frac{Ebt_{1}}{l_{1}},\;K_{l2}=\frac{Ebt_{2}}{l_{2}},\;K_{\theta 1}=\frac{Ebt_{1}^{3}}{12l_{1}},\;K_{\theta 2}=\frac{Ebt_{2}^{3}}{12l_{2}}.$$

If the condition of *F_out_* = 0 is assumed, the amplification ratio and input stiffness of the bridge-type amplification mechanism can be expressed as [50]:

(20) $$R_{amp}=\frac{2x_{out}}{2x_{in}}=\frac{C_{21}}{C_{11}},$$

(21) $$K_{in}=\frac{F_{in}}{x_{in}}=\frac{2}{C_{11}}.$$

When *F_in_* = 0, the output stiffness of the bridge-type amplification mechanism is formulated as:

(22) $$K_{out}=\frac{F_{out}}{2x_{out}}=\frac{1}{C_{22}}.$$

Therefore, the amplification ratio model of the bridge-type amplification mechanism can be expressed as follows:

(23) $$R_{amp}=\frac{2x_{out}}{2x_{in}}=\frac{w(l_{1}+l_{2})\left(2/K_{\theta 1}+1/K_{\theta 2}\right)}{8/K_{l1}+4/K_{l2}+w^{2}\left(2/K_{\theta 1}+1/K_{\theta 2}\right)}.$$

### 3.3. Stiffness Model of the Positioning Stage

**Figure 10:**
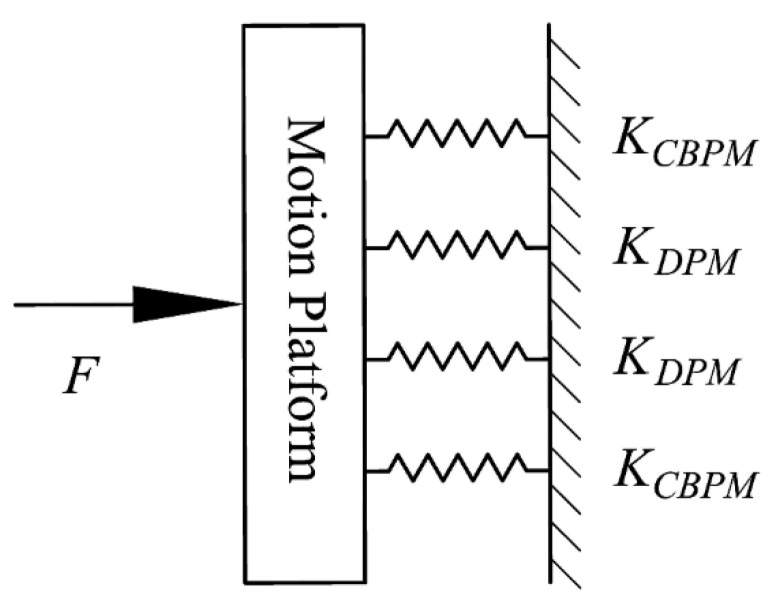
Equivalent stiffness model of the motion platform.

According to the parallel relationship among the bridge-type amplification mechanism, compound parallelogram mechanism, double parallelogram mechanism, and motion platform in the precision positioning stage, the equivalent stiffness model of a single motion platform moving in the X-axis (similarly for the Y-axis) is shown in Figure 10. The compound parallelogram mechanisms, C_3_ and C_4_, and the double parallelogram mechanisms, D_1_ and D_2_, are all connected in parallel with the motion platform. As a result, the input stiffness of a single motion platform is expressed as:

(24) $$K_{load}=2(K_{CBPM}+K_{DPM}).$$

If the motion platform is regarded as the elastic load of the bridge-type amplification mechanism, the stiffness of the elastic load is *K_load_*, and the loading force can be obtained as

(25) $$F_{load}=K_{load}\cdot X_{stage},$$

where *X_stage_* is the output displacement of the positioning stage. The introduction of the loading force causes the input and output ends of the bridge-type amplification mechanism to generate displacements opposite to the intended direction of motion. Therefore, the relationship between the input force and the output displacement of the entire positioning stage is different from that of the bridge-type amplification mechanism at no-loading status; hence, further analysis is required. The displacement of the input and output ends of the bridge-type amplification mechanism under the actuation of input force *F_in_* alone can be defined as *x_in_*_1_ and 2*x_out_*_1_, respectively. Similarly, the displacement of the input and output ends under the actuation of loading force *F_load_* alone can be defined as *x_in_*_2_ and 2*x_out_*_2_, respectively. The actual displacement of the input and output ends are presented as:

(26) $$X_{in}=x_{in1}-x_{in2},$$

(27) $$X_{stage}=2x_{out1}-2x_{out2},$$

where,

$$x_{in1}=\frac{F_{in}}{K_{in}},\;x_{out2}=\frac{F_{load}}{2K_{out}}.$$

According to equation (18), the following equations are derived:

(28) $$x_{out1}=x_{in1}\cdot R_{amp},$$

(29) $$x_{in2}=x_{out2}/R_{amp}.$$

The relationship between the actual input displacement and the output displacement of the positioning stage is shown below:

(30) $$X_{stage}=2X_{in}\cdot R_{amp}.$$

Substituting Equations (25)–(29) into Equation (30), the relationship between input force and output displacement of the entire positioning stage can be derived:

(31) $$F_{in}=\frac{K_{in}(K_{out}+K_{load})}{2K_{out}\cdot R_{amp}}\cdot X_{stage}.$$

Substituting Equation (30) into Equation (31) and eliminating *X_stage_*, the input stiffness of the entire positioning stage is calculated as:

(32) $$K_{in}^{stage}=\frac{K_{in}(K_{out}+K_{load})}{K_{out}}.$$

Simultaneously, due to the parallel structure of the motion platform and the bridge-type amplification mechanism, the output stiffness of the entire positioning stage is finally obtained:

(33) $$K_{out}^{stage}=K_{out}+K_{load}.$$

## 4. Finite Element Simulation

The finite element analysis of the precision positioning stage was carried out by the finite element software ANSYS 18.2. The material was chosen to be Al7075 with Young’s modulus of 71.7 GPa, Poisson’s ratio of 0.33, yield strength of 503 MPa, and density of 2810 kg/m^3^. The main dimensional parameters of the stage are shown in Table 2, where *t*, *L_DPM_*, and *L_CBPM_* are the thickness and length of flexible beams in double parallelogram mechanisms and compound parallelogram mechanisms, respectively.

### 4.1. Static Analysis

**Figure 11:**
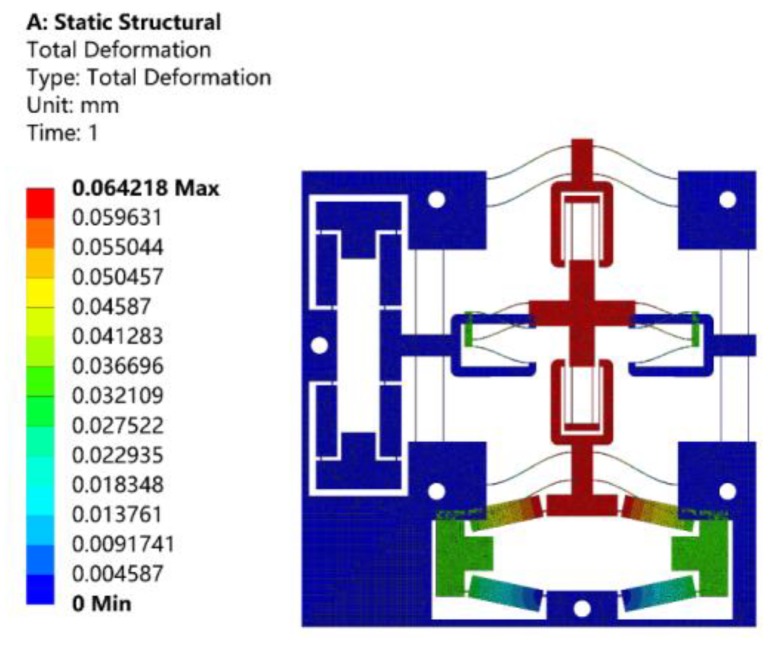
Analysis of the displacement amplification ratio by ANSYS.

Static analysis was performed to verify the prediction of the deformation of the proposed stage, input and output stiffness, and displacement amplification ratio of the proposed theoretical model. When a total displacement of 10 μm was applied to the bridge-type amplification mechanism in the X-axis, the resulting motion of the stage in the Y-axis is shown in Figure 11. As can be observed from Figure 11, the displacement of the stage was 64.2 μm, and the amplification ratio was about 6.42. According to Equation (16), the theoretical amplification ratio was calculated as 6.57, and the relative error was 2.34%, compared to the finite element value.

**Figure 12:**
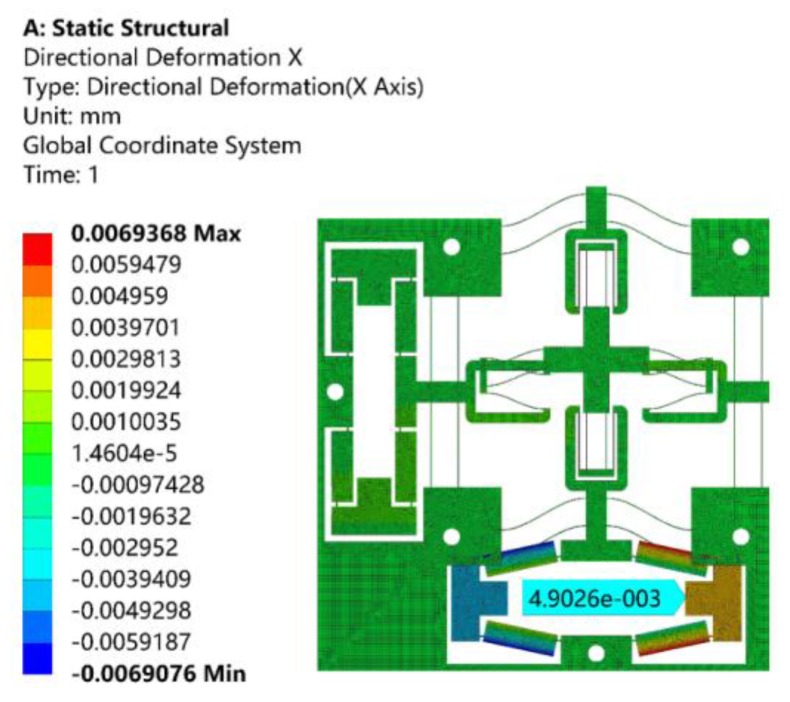
Analysis of the input stiffness by ANSYS.

When a driving force of 10 N along the X-axis was, respectively, applied to the input ends of bridge-type amplification mechanism, the resulting displacements of the input ends are shown in Figure 12. As can be observed from Figure 12, the displacement of the input end along the X-axis positive direction was 4.9 μm, and the input stiffness was about 2.039 N/μm. The theoretical input stiffness calculated by Equation (32) was 2.118 N/μm, and the relative error was 3.87%.

**Figure 13:**
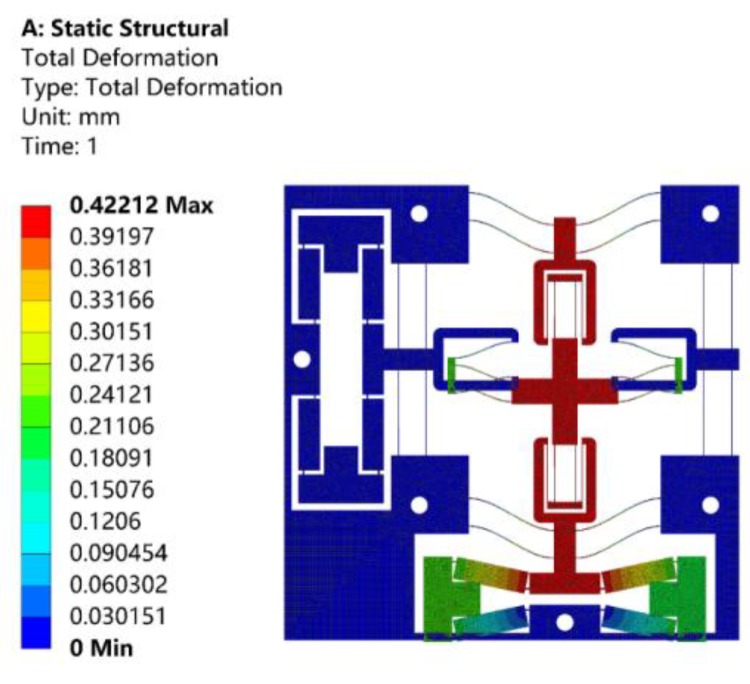
Analysis of the output stiffness by ANSYS.

When a 10 N driving force along the negative direction of the Y-axis was applied to the output end of the positioning stage, the resulting output displacement is shown in Figure 13. As can be observed from Figure 13, the displacement of the output end was 422.1 μm, and the output stiffness was about 23.69 N/mm. The theoretical output stiffness calculated by Equation (33) was 24.32 N/mm, and the relative error was 2.66%. It should be noted that, since the displacement of the stage was a micron, the lateral stiffness of the compound parallelogram mechanisms in the calculation can be approximated as:

(34) $$K_{CBPM}=\left(48+\frac{2.88\delta_{y}^{2}}{1/d+\delta_{y}^{2}/700}\right)\cdot \frac{EI}{L^{3}}\approx \frac{48EI}{L^{3}}.$$

**Figure 14:**
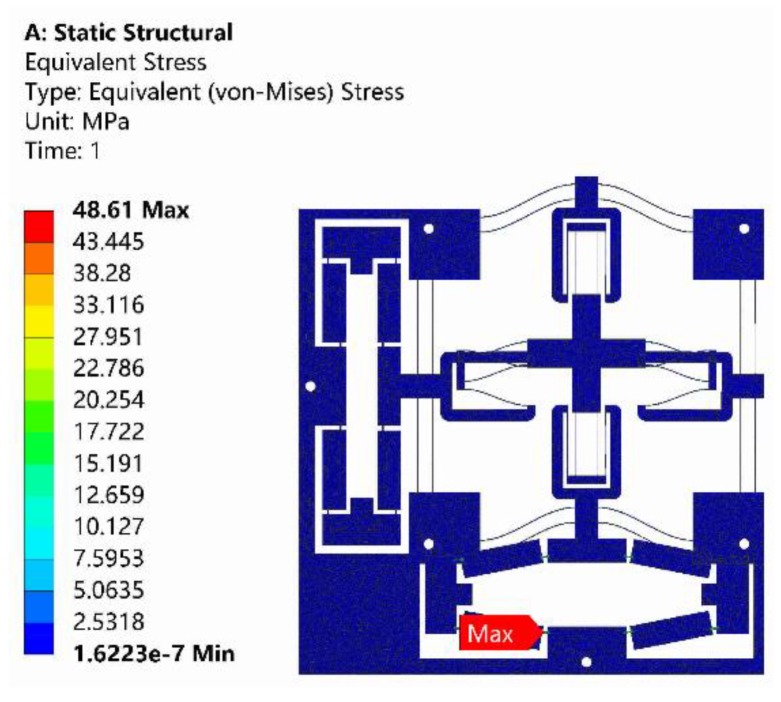
Analysis of the maximum stress by ANSYS.

The adopted PEAs had an actual stroke of 65 μm. Therefore, the input displacement of 65 μm was applied to the bridge-type amplification mechanism along X-axis, and the resulting maximum stress is shown in Figure 14. As can be observed from Figure 14, the maximum stress of 48.61 MPa occurred at the surface of flexure hinge, which was far below the yield strength of Al7075.

**Figure 15:**
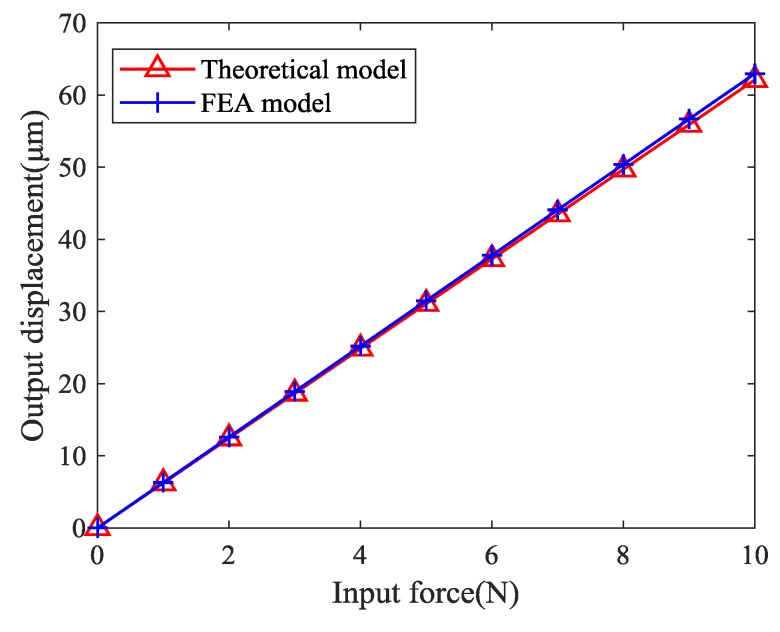
Input forces and output displacements of the stage.

Performances of the positioning stage are listed in Table 3 and the relationship between the output displacement and the input force of the positioning stage is presented in Figure 15. The maximum error of the theoretical model and the finite element model was less than 4%, which verifies the correctness of the theoretical model.

### 4.2. Dynamic Analysis

**Figure 16:**
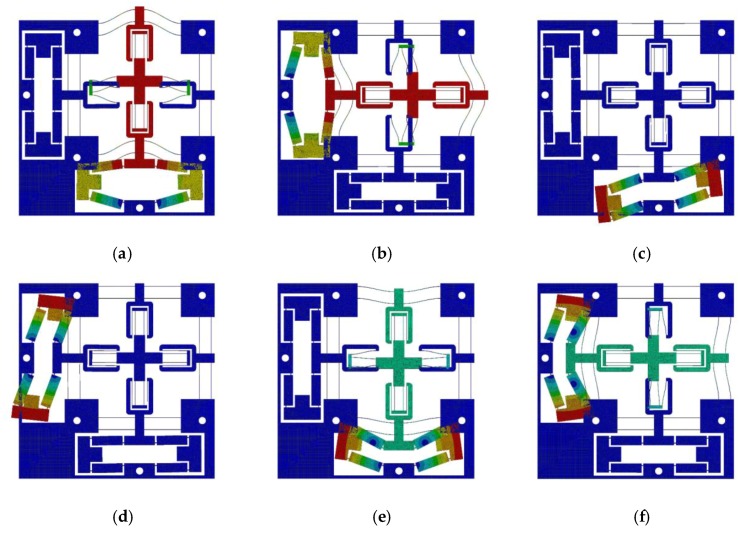
Modal analysis of the stage by ANSYS: (**a**) 44.234Hz, (**b**) 44.335Hz, (**c**) 88.232Hz, (**d**) 88.393Hz, (**e**) 98.418Hz, and (**f**) 98.539Hz.

In order to investigate the dynamic characteristics of the proposed positioning stage, a modal analysis was performed by the ANSYS Workbench and the first six modes were obtained, as shown in Figure 16. The first and fifth modes were the stage moving along Y-axis, the second and sixth modes were the stage moving along X-axis, and the first six modes were X–Y in-plane modes. Since the structure of the positioning stage was completely symmetrical, deformations in the X-axis and the Y-axis were basically similar, and the natural frequencies were very close. The first and second resonant frequencies were 44.234 Hz and 44.335 Hz, respectively.

## 5. Experiment Setup

**Figure 17:**
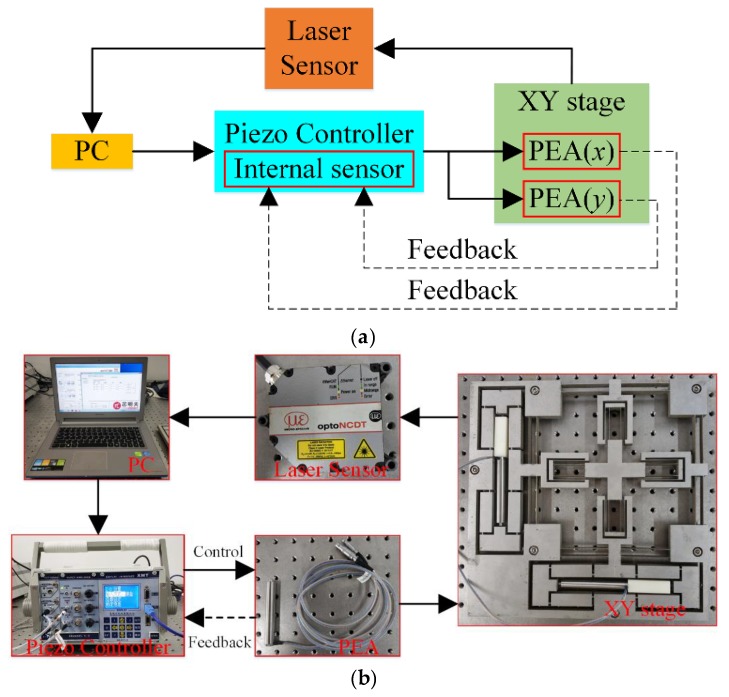
Experimental setup of the positioning stage: (**a**) Schematic diagram, and (**b**) photograph of the experimental setup.

The experimental setup is established in Figure 17. A prototype of the stage was fabricated by the wire electrical discharge machining (WEDM) process with a piece of Al7075. Two PEAs (PSt150/7/80 VS12 from COREMORROW, Inc., Harbin, China), pre-tightened by an appropriate screw, were installed into the bridge-type amplification mechanisms to actuate the stage; the technical parameters of the PEA are listed in Table 4. A piezo controller (E01.D3 from COREMORROW, Inc., Harbin, China) which realized closed-loop control through the inside position sensor was utilized to drive the PEAs, and the influence of the hysteresis characteristics of PEA were almost eliminated. The output displacement and cross-coupling error of the stage were measured by a laser sensor (optoNCDT2300 from MICRO-EPSILON Messtechnik GmbH, Ortenburg, Germany) with a 2 mm measurement range and 30 nm resolution. 

**Figure 18:**
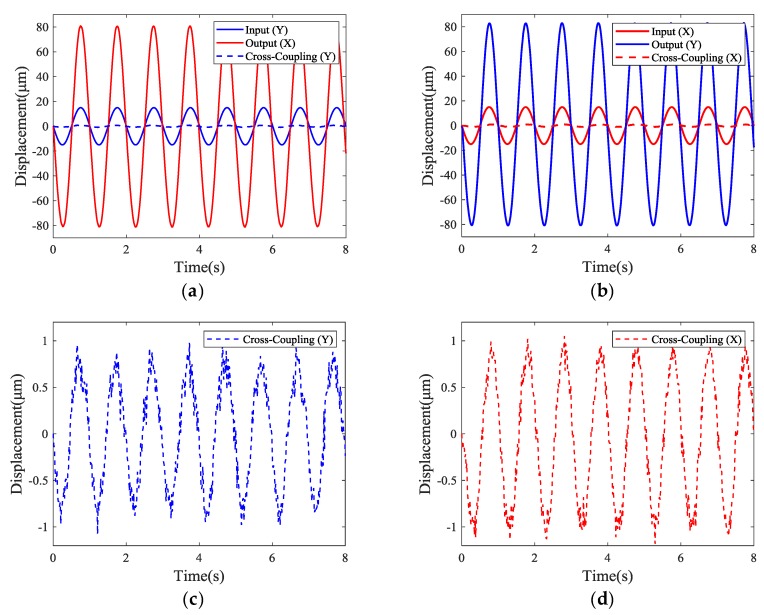
Experimental performance of the stage in the X-axis: (**a**) Output displacement under Y-axis actuation, (**c**) Cross-coupling error in Y-axis; as well as performance in the Y-axis: (**b**) Output displacement under X-axis actuation, (**d**) Cross-coupling error in the X-axis.

First, a 1 Hz sinusoidal command signal with peak-to-peak amplitude of 30 μm was applied to the PEA to test the amplification ratios and cross-coupling errors. The experimental results, including the output displacements and cross-coupling errors were measured by the laser sensor in both directions, which are shown in Figure 18. The maximum displacements in the X-axis and Y-axis of the stage were 80.89 μm and 82.70 μm, respectively, which indicates that the amplification ratios of the stage in the X-axis and Y-axis were 5.39 and 5.51, respectively. Thus, the experimental amplification ratios in the X-axis and Y-axis were 19.1% and 16.5% smaller than the FEA results, respectively. These errors were mainly due to the fact that the tip of the PEA was a ball head and, hence, the contact area between the spherical surface and the plane made the real contact surface in the experiment smaller than the size set in the finite element simulation, thus leading to a deviation. In addition, Figure 18 also shows the cross-coupling errors in the Y-axis (X-axis) when the stage moved along the X-axis (Y-axis). As depicted in Figure 18, the maximum cross-coupling errors in the Y-axis and X-axis were approximately 1.1 μm and 1.2 μm, respectively. Hence, the experimental cross-coupling errors in the Y-axis and X-axis were 1.36% and 1.45%, respectively. The difference in performance between the two axes may be mainly attributed to: (1) The asymmetry of the stage due to manufacturing errors, (2) the different pre-loading conditions of the PEAs, and (3) the influence of the contact area on one side of the input. 

**Figure 19:**
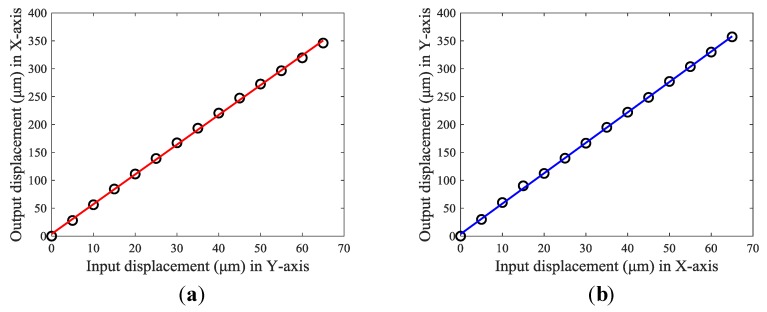
Experimental testing results of the stroke in (**a**) output displacement in the X-axis and (**b**) output displacement in the Y-axis.

Second, the workspace of the positioning stage was tested by applying several discrete input displacements to the PEAs. The testing results are illustrated in Figure 19. It can be observed that the output displacements of the stage increased linearly with an increase of the input displacements. However, when the output displacements became constant and no longer changed with the increase of input displacements, it meant that the blocked force of the PEA was reached. Even if a larger driving displacement was applied in the controller software under this circumstance, the actual output displacements of the PEA remained unchanged, causing the output displacement of the stage to remain constant. In such a case, the maximum output displacement of the PEA was 65 μm and the maximum output displacements of the stage in the X-axis and Y-axis were 346.1 μm and 357.2 μm, respectively. Thus, the tested workspace of the stage was 346.1 × 357.2 μm.

## 6. Conclusions

In this paper, a decoupled compliant positioning stage was designed, based on bridge-type amplification mechanisms and parallelogram mechanisms. Two kinds of parallelogram mechanisms and a bridge-type amplification mechanism were modeled by a Beam constrained model and Castigliano’s second theorem, respectively. Based on this, the displacement amplification ratio, input stiffness, and output stiffness of the entire stage were derived, and the results of theoretical model were verified by finite element analysis. Compared with the finite element results, the theoretical model had an error of 2.34% in the amplification ratio, 3.87% in the input stiffness, and 2.66% in the output stiffness, which further validated the proposed modeling method. The prototype was fabricated to test the amplification ratio, cross-coupling error, and workspace. The experimental results demonstrated that the stage had a relatively large workspace, of 346.1 × 357.2 μm, with a corresponding amplification ratio of 5.39 in the X-axis and 5.51 in the Y-axis, while the cross-coupling error was less than 1.5%.

## Figures and Tables

**Table 1:** Calculation parameters of beam constraint model.

Parameters	Values	Parameters	Values
*a*	12	*i*	−0.6
*b*	4	*j*	−1/15
*c*	−6	*k*	1/20
*e*	1.2	*r*	1/700
*g*	2/15	*s*	11/6300
*h*	−0.1	*q*	−1/1400

**Table 2:** Structural parameters of positioning stage.

Parameters	Values	Parameters	Values	Parameters	Values
*t*	0.5	*l*_1_	3	*L_CBPM_*	60
*t*_1_	1	*l*_2_	50	*w*	8
*t*_2_	15	*L_DPM_*	40	*b*	12

**Table 3:** Performance of the proposed positioning stage.

Model	*R_amp_*	$K_{in}^{stage}\;(N/\mu m)$	$K_{out}^{stage}\;(N/\mathrm{mm})$
Analytical	6.57	2.118	24.32
FEA	6.42	2.039	23.69
Error	2.34%	3.87%	2.66%

**Table 4:** Technical parameters of the PEA.

Properties	Values
Nominal displacement (μm)	76 ± 10%
Stiffness (N/μm)	12 ± 20%
Blocked force (N)	1200
Capacitance (μF)	7.2 ± 20%
Resonant frequency (kHz)	12
Dimensions (mm)	Φ 12 × 87.5
